# An amygdala-cortical circuit for encoding generalized fear memories

**DOI:** 10.1038/s41380-025-03140-8

**Published:** 2025-08-12

**Authors:** Carly J. Vincent, Ricardo Aguilar-Alvarez, Samantha O. Vanderhoof, David D. Mott, Aaron M. Jasnow

**Affiliations:** 1 Department of Physiology, Pharmacology, and Neuroscience, University of South Carolina School of Medicine, Columbia, SC 29209 USA; 2 Department of Psychological Sciences, Brain Health Research Institute, Kent State University, Kent, OH 44242 USA

**Keywords:** Neuroscience, Psychology, Physiology

## Abstract

Generalized learning is a fundamental process observed across species, contexts, and sensory modalities that enables animals to use past experiences to adapt to changing conditions. Evidence suggests that the prefrontal cortex (PFC) extracts general features of an experience that can be used across multiple situations. The anterior cingulate cortex (ACC), a region of the PFC, is implicated in generalized fear responses to novel contexts. However, the ACC’s role in encoding contextual information is poorly understood, especially under increased threat intensity that promotes generalization. Here, we show that synaptic plasticity within the ACC and signaling from basolateral amygdala (BLA) inputs during fear learning are necessary for generalized fear responses to novel encountered contexts. The ACC did not encode specific fear to the training context, suggesting this region extracts general features of a threatening experience rather than specific contextual information. Together with our previous work, our results demonstrate that generalized learning about threatening contexts is encoded, in part, within an ascending amygdala-cortical circuit, whereas descending ACC projections to the amygdala drive generalized fear responses during exposure to novel contexts. Our results further demonstrate that schematic learning can occur in the PFC after single-trial learning, a process typically attributed to learning over many repeated learning episodes.

## Introduction

To survive, animals must use experience to adapt flexibly to changing and uncertain conditions. Animals typically use generalization as an adaptive process to assess if new objects or situations are likely to produce the same outcome as those previously experienced. Thus, unlike a lack of discrimination, which is the failure to detect a difference between two stimuli, generalization is an active cognitive process that promotes appropriate behavioral action across contexts and stimuli [1]. Likewise, generalizing fear responses is evolutionarily adaptive because it supports survival by enabling animals to avoid threatening situations similar to ones experienced previously but come at the cost of missing out on food resources or access to mates. In fear conditioning protocols, generalization occurs when animals, including humans, produce fear responses to contexts or cues that are similar but distinctly different than those previously associated with an aversive stimulus; this is the basis for the overgeneralization observed with anxiety and stress-related disorders [e.g., post-traumatic stress disorder (PTSD)] [2, 3]. Indeed, fear generalization is recognized as a transdiagnostic mechanism for anxiety disorders [4]. Studies report that increasing threat intensity (e.g., shock) results in more generalization in rodents [5] and humans [6, 7], but how fear spreads across multiple stimuli during increased threat is not well-established [8]. Furthermore, whereas valuable mechanisms related to encoding and expressing fear discrimination to extensively trained discriminatory contexts and tones (i.e., CS^+^ and CS^-^) have been identified [8–13], how rapid encoding for generalizing to never-experienced stimuli or circumstances after single-trial learning are not well defined.

The medial prefrontal cortex (mPFC) extracts general features of an experience rather than detailed contextual or spatial information [9, 14]. The anterior cingulate cortex (ACC, Brodmann’s area 24A, 24B), a subregion of the mPFC, is involved in the expression of generalized fear memories [5, 15–17] and storing remote memories [18–20] but its role in encoding context representations is less clear. The nucleus reuniens (RE) is a thalamic structure that connects the PFC to the hippocampus and is important in acquiring hippocampal-dependent contextual memories [21, 22]. Without a functioning RE, acquired memories are hippocampal-independent and presumably rely on cortical regions to be encoded [22]. However, even with a fully functioning RE, the mPFC supports generalized representations, suggesting it may operate in parallel with an RE-hippocampal network to encode different task features [9, 14]. The basolateral amygdala (BLA) sends prominent projections to the mPFC [23, 24], but how these inputs to the ACC are involved in encoding context and threat information is poorly understood, especially under conditions that promote generalized memories [5–7]. To examine these questions, we used pharmacological and chemogenetic-mediated circuit manipulations to uncover the role of the ACC in encoding highly salient experiences that generalize across contexts.

## Methods & materials

### Animals

Experiments used 234 male and female mice or male mice only. Details regarding sample sizes are reported in the results section for each experiment. Animals were F1 129B6 hybrids derived from crossing C57BL/6J males with 129S1/SvImJ females, between the age of 7–12 weeks old. All mice were generated in a breeding colony at the University of South Carolina School of Medicine. All mice were housed in groups of two to five per cage, had ad libitum access to food and water, and were maintained on a 12:12 light-dark cycle.

### Ethics approval

All procedures were conducted in a facility accredited by the American Association for Laboratory Animal Care (AALAC), per the National Institutes of Health guidelines, and with approval by the University of South Carolina Institutional Animal Care and Use Committee (IACUC), and maintained with adherence to the National Research Council’s Guide for the Care and Use of Laboratory Animals. None of the experiments were conducted blinded, and all animals were randomly assigned to treatment groups.

### Surgeries

#### Cannulations

Mice were given a subcutaneous injection of Rymadil (2.5 mg/kg) prior to anesthesia. Following drug administration, mice were anesthetized with inhaled isoflurane (3% induction: 1–1.5% maintenance). Mice were then tested for lack of motor responses before placement into the stereotaxic apparatus (David Kopf Instruments, Tujunga, CA). An incision on the scalp was made and measurements of lambda and bregma landmarks were determined. For the pharmacology experiments, a unilateral burr hole was drilled using the following coordinates for the ACC: (+0.80 mm AP, +0.70 mm ML, −1.75 mm DV). A 26-gauge stainless steel cannula with a 4 mm pedestal (Plastics One, Inc.) set at a 14° angle was guided to the appropriate position in relation to bregma using the stereotaxic apparatus. For the circuit activation experiment, two guide cannula (Plastics One) were surgically implanted bilaterally above the BLA (−1.6 mm AP, ±3.4 mm ML, −4.9 mm DV from bregma). Dummy cannula were inserted into the guide cannula after surgery. Following surgery, mice were removed from the apparatus, placed into a clean cage, and monitored until fully awake.

#### Virus infusions

All mice were given a subcutaneous injection of Rymadil (2.5 mg/kg) prior to anesthesia. Following drug administration, mice were anesthetized with inhaled isoflurane (3% induction: 1–1.5% maintenance). Mice were then tested for lack of motor responses prior to placement into the stereotaxic apparatus (David Kopf Instruments, Tujunga, CA). An incision on the scalp was made and measurements of lambda and bregma landmarks were determined. Bilateral burr holes were drilled using the following coordinates for the Prelimbic Cortex (PL): (+ 1.90 mm AP, +0.60 mm ML, −2.25 mm DV) at a 12° angle; Anterior Cingulate Cortex (ACC): (+0.80 mm AP, +0.70 mm ML, −1.75 mm DV) at a 14° angle; BLA (−1.6 mm AP, +3.4 mm ML, −4.9 mm DV). All infusions were completed using 2 μl Neuros syringes (Hamilton, Reno NV) with 30-gauge needles. For PL inactivation, pAAV-hSyn-hM4D(Gi)-mCherry (AAV8) (Addgene) or pAAV-hSyn-EGFP (AAV8) (Addgene) were bilaterally infused into the PL at a volume of 0.2 µl per side, at a rate of 0.1 µl per minute. For BLA-ACC circuit inactivation, a retrograde pAAV-Ef1a -cre (AAVrg) (Addgene) was bilaterally infused into the ACC at a volume of 0.6 µl per side at a rate of 0.2 µl per minute [25–27]. Following the ACC infusion, pAAV-hSyn-DIO-hM4D(Gi)-mCherry (AAV8) or pAAV-hSyn-DIO-mCherry (AAV8) (Addgene) was bilaterally infused into the BLA at a volume of 1.0 µl per side at a rate of 0.2 µl per minute. For the BLA-ACC circuit activation experiment, a retrograde adeno-associated virus (pAAV-hSyn-hM3Dq-mCherry (AAVrg) or pAAV-CaMKIIa-EGFP (AAVrg)) was infused into the ACC at a volume of 0.2 µl per side at a rate of 0.1 µl per minute [5]. Five weeks later mice were cannulated bilaterally over the BLA and allowed 1 week to recover prior to behavioral testing. This enabled the activation of BLA neurons projecting to the ACC, similar to our previous methods [5].

### Behavioral procedures

#### Context fear conditioning

Mice were trained using contextual fear conditioning in four identical conditioning chambers (12″ W × 12″ D × 12″H) containing two Plexiglass walls, two aluminum sidewalls, and a stainless-steel grid-shock floor (Colbourn Instruments, Allentown, PA). The standard training context (Context A) consists of the conditioning chamber with a polka-dot insert attached to the rear Plexiglass wall, white noise (70db), dim illumination, and the stainless-steel grid floors cleaned with 70% ethanol. Mice received two days of pre-exposure for 5 min. Then, the following day, the mice underwent nine minutes of fear conditioning by pairing the training context with a series of 5 un-signaled foot shocks (1.0 mA) separated by 90-s inter-shock intervals (ISI). Measures of freezing were recorded using the Freezeframe5 (Actimetrics). Mice were then tested for fear expression 24-h after training by examining freezing across the testing period consisting of a 5-min exposure to the training context (Context A) and a novel context (Context B) in a counterbalanced design with 72 h between tests. We have previously demonstrated that this procedure produces no order effects [5]. The novel context had no background element, a flat floor, a fan (60db), infrared (IR) lights and was cleaned with 2% quatricide (Pharmacal). For experiments that assessed activity-regulated cytoskeletal-associated protein (Arc) expression in the ACC, one group of mice was exposed to an immediate shock procedure to take advantage of the immediate shock deficit [28, 29]. When rodents are shocked immediately after being placed into a conditioning context, they do not exhibit freezing upon re-exposure, presumably because they have not had enough time to develop a representation of the conditioning context [30]. Before immediate shock, mice received two days of pre-exposure, each for five minutes, to a novel cage to control for pre-exposure in the group of mice receiving context fear conditioning. Then, the following day, mice underwent the immediate shock procedure by being placed in the training context and were administered 5 un-signaled foot shocks in 10 s (1.0 mA). After the last shock, mice were immediately removed from the chamber and returned to their cage.

#### Immediate shock and alternative context training procedure

To demonstrate the involvement of associative learning in generalization, we utilized an immediate shock procedure, which limits the ability of the mice to form a representation of the training context [31]. The immediate shock procedure was conducted as previously described.

Mice should not freeze to all contexts in which they are placed after having undergone context fear-conditioning. To demonstrate that mice show context generalization due to similarities in the contextual elements between the training and novel contexts, we trained the mice in an alternative training context (Context C) that differed substantially from the standard training conditions. For the alternative training context procedure, mice were fear-conditioned in the alternative conditioning chamber (Context C) and then exposed to our standard training context (Context A) six hours later without shock exposure, similar to the exposure in the immediate shock procedure above. Mice were tested for fear in the novel (Context B) and standard training (Context A) contexts as above. The alternative training context (Context C) consisted of operant conditioning chambers that differed substantially in size and contextual cues from our standard training context (Context A) (Med-Associates; 8.25″ W × 7.5″ D × 11″ H), with a black and white striped insert on the rear wall, a vanilla-scented cotton ball placed in the bottom tray of the chamber, and stainless-steel grid floors that were cleaned with 70% ethanol prior to and in between conditioning. Two nose pokes and a food/liquid reward trough were also present, but neither was activated during the training. Mice were conditioned in the dark, and the training conditions were 5 un-signaled foot shocks (1.0 mA) separated by 90-s inter-shock intervals (ISI). Mice were then exposed to the standard training context for 9 min, the same duration as the fear-conditioning procedure, 6 h later. The following day, mice were tested in the standard training context (Context A) or a novel context (Context B) for 5 min. Mice were assessed in the standard training context (Context A) to test for the specific unpairing of the shocks from the context, whereas mice were tested in our novel context (Context B) to demonstrate a lack of contextual overlap. Mice underwent testing 72 h after the first test in the context that was opposite to the one in which they were originally tested. Additionally, to assess fear to the alternative training context (Context C) mice underwent a 5-m inute test, 6 days after the last exposure (Day 13). Freezing was recorded using the Freezeframe5 (Actimetrics), or ANY-Maze 4.99 software during training and testing in the alternative training context (Context C) (Stoelting, Wood Dale, IL).

#### Open field test

For the assessment of BLA-to-ACC circuit inactivation on locomotor behavior, mice were placed in four identical 45 × 45 cm white acrylic open-field arenas. Lighting was adjusted to 10–12 lux. Each arena was cleaned with 10% ethanol prior to and in between uses. Mice were recorded for 10 min, and distance traveled was assessed using Ethovision15 software.

### Drug preparation

Lidocaine HCL (Sigma) was dissolved in phosphate-buffered saline at a concentration of 4% w/v and adjusted to a pH of 7.0 [15, 19]. DL-AP5 (HelloBio) was dissolved in 0.9% saline at a concentration of 45 mM (2.7 μg/side) [16, 32, 33]. Clozapine-N-oxide dihydrochloride (CNO) (HelloBio) was dissolved in 0.9% saline at a dose of 5 mg/kg, for intraperitoneal injections, or 0.9% saline at a concentration of 650 μM for intracranial infusions [5].

### Intracranial infusions

For all intracranial pharmacological experiments, mice received infusions immediately before or after contextual fear conditioning. Mice were transported from the animal colony to a room adjacent to the fear conditioning room, where infusions occurred. For post-training infusions, mice were removed from the fear conditioning chambers, placed into their home cage, and transported to the infusion area. For intracranial infusions, mice were placed in a clean cage, and a 33-gauge internal infusion needle attached to polyethylene tubing (PE-20) was inserted into the guide cannula. A 5 μL Hamilton syringe attached to a micro-infusion pump from Harvard Apparatus was used to infuse the drug or vehicle solution. All mice received a 0.2 μL infusion of vehicle or drug unilaterally into the ACC at a rate of 0.1 μL/min. For BLA infusions, mice received 0.2 μL (per side) of CNO bilaterally into the BLA at a rate of 0.1 μL/min. After the infusions, needles were left in place for 2 min to allow for diffusion of the drug. Afterward, dummy cannulas were inserted, and mice were returned to their home cage or conditioning chambers.

### Cannulation site verification

After behavioral testing, mice were infused with 4% fluro-gold (Flurochrome, LLC) at 0.05 μL/min for a total volume of 0.1 μL via guide cannula. Brains were extracted and stored in a −80 °C freezer until slicing. Sections were sliced at 40 μm on a cryostat (Microm HM 500) and placed onto Superfrost™ Plus slides. Sections were cover-slipped with prolonged diamond mounting media, and sites were examined using an epi-fluorescent microscope (Leica). Sites that were outside of the ACC or BLA or animals that failed to infuse during the behavioral procedure were excluded from statistical analyses.

### Immunohistochemistry

To verify virus expression after behavioral testing, mice were deeply anesthetized and transcardially perfused using 0.9% saline, followed by 4% paraformaldehyde. Brains were post-fixed for 24 h, then transferred to 30% sucrose solution until fully submerged. Brains were sliced on a freezing microtome (Leica) at 40 μM thickness and stored in an ethylene glycol/sucrose antifreeze solution at −20 °C until processing. Sections were then stained for mCherry using immunohistochemistry. All sections were incubated in hydrogen peroxide (0.08 then 0.3%), washed in PBS, and then incubated in rabbit anti-mCherry primary antibody (1:30,000; abcam, ab167453) for 1 h at room temperature then 4 °C for 48 h with constant agitation. After primary antibody incubation, tissue was washed in PBS, then incubated with a goat anti-rabbit biotinylated secondary antibody (1:500; Jackson Immuno, 111-005-003) for 1 h at room temperature. After, avidin-biotin complex (ABC; Vector Laboratories) was applied to the tissue for 1 h. Following ABC application, tissue was washed briefly in PBS and sodium acetate (0.175 M) and visualized by adding Ni-enhanced 3, 3′- Diaminobenzidine (DAB). Ni-DAB solution was applied for 20 min. Following Ni-DAB enhancement, tissue was washed in sodium acetate (0.175 M) and PBS. Afterward, the tissue was mounted and dehydrated via increasing changes of ethanol and the application of xylenes for clearing. Sections were cover slipped using DPX mountant and visualized on a light microscope. After immunohistochemical detection, virus expression was imaged on a light microscope, and viral spread was transcribed to the mouse stereotaxic atlas. Exclusion criteria included sites that contained hM4Di-mCherry expression outside of the BLA and a lack of fiber staining within the ACC. Expression patterns were categorized into ‘minimum,’ ‘representative,’ and ‘maximum’ spread. The ‘minimum’ spread was the minimum spread of virus observed across all mice in the group and included in the statistical analysis. The ‘maximum’ spread was the maximum spread of virus observed across all mice and included in the statistical analysis. The ‘representative spread’ was the average spread of virus that was observed in a particular experiment.

For immunohistochemical detection of activity-regulated cytoskeletal-associated protein (Arc), mice were perfused 60 min after context fear conditioning. Brains were post-fixed for 24 h, then transferred to 30% sucrose solution until fully submerged. Brains were sliced on a freezing microtome (Leica) at 40 μM thickness and stored in an ethylene glycol/sucrose antifreeze solution at −20 °C until processing. Sections were removed from cryoprotectant solution and washed in PBS. Following washes, sections were incubated in rabbit anti-Arc primary antibody (1:30,000; abcam, ab183183) for 1 h at room temperature, then 4° for 48 h with constant agitation. Following incubation, sections were washed with PBS and incubated in a goat anti-rabbit biotinylated secondary antibody (1:500; Jackson Immuno, 111-005-003) for 1 h at room temperature. Following incubation, sections were washed and placed in Avidin-Biotin Complex (ABC), and washed and enhanced with Ni-DAB, as described above. Afterward, tissue was mounted and dehydrated via increasing changes of ethanol and application of xylenes for clearing. Sections were then coverslipped using DPX mountant, then visualized on a light microscope.

### Analysis of Arc expression

To assess Arc protein expression in the ACC, images were taken on a light microscope (Leica DM 500B) using a 20X objective. Images were analyzed using FIJI (NIH Image) to set a region of interest (ROI) for analysis. The ROI was 200 × 200 μm section set in the ACC. Landmarks were used to guide consistency in placement across slices [34] per the mouse brain atlas [35]. Four non-consecutive sections were analyzed by manually counting cells within the ROI [34]. Researchers were blinded to treatment conditions throughout the counting process.

### Generalization index analysis

Generalization indexes were calculated in experiments that utilized within-subjects testing to show difference scores for freezing in the training and novel contexts. The absolute value of the [novel context freezing – training context freezing] was determined and divided by the sum of the [novel context freezing + training context freezing]. Scores then ranged from 0–1. A score of 0 indicates no difference in freezing between the training and novel contexts, and a score of 1 indicates a maximum difference in freezing between the two contexts.

### Statistical analyses

All data were analyzed using GraphPad Prism statistical software (GraphPad 10) using unpaired t-tests, between subjects, or repeated measures two-way Analysis of Variance (ANOVA). G*Power 3 was used to determine if experiments were appropriately powered for each experiment. Outliers were removed using 2 standard deviations method. Statistically significant ANOVAs were followed with Tukey’s or Sidak’s post-hoc analyses. Please refer to Tables 1 and 2 in the supplemental materials for statistical details.

## Results

### Plasticity in the anterior cingulate cortex is engaged during strong context fear conditioning

**Fig. 1:**
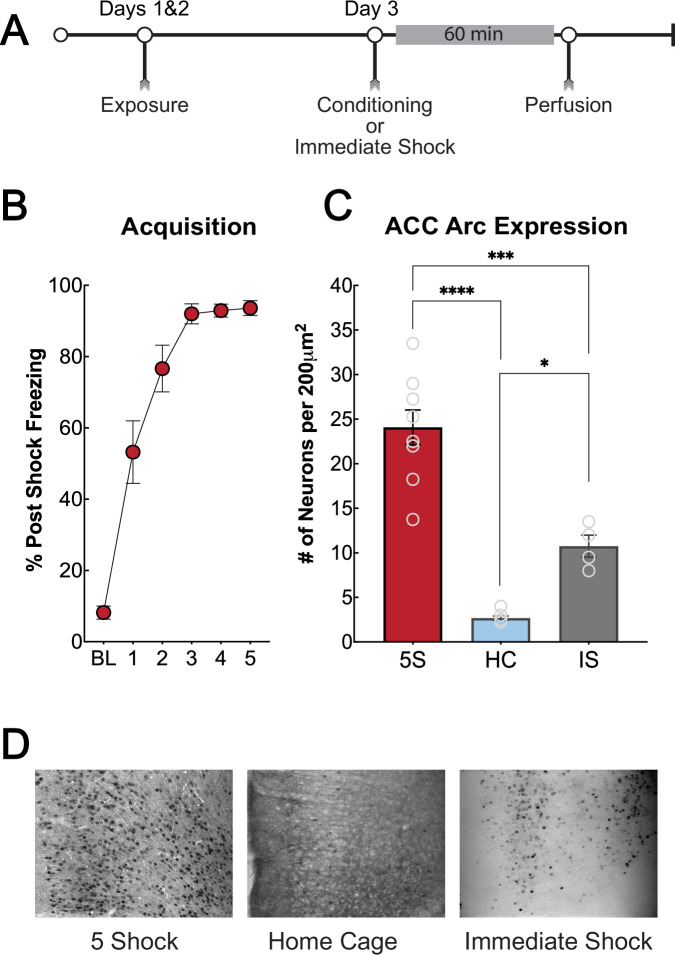
Arc expression in the ACC is increased during strong context fear conditioning. **A**: Timeline for behavior and Arc immunohistochemistry experiment. Mice underwent contextual fear conditioning as described. Sixty minutes after training, mice were perfused, and brain tissue was processed for immunohistochemistry to assess the expression of the plasticity-associated protein Arc. **B**: Post-shock freezing during acquisition increased after successive presentations of the 5 un-signaled foot shocks (*p* < 0.0001). **C**: Quantification of Arc expression in the ACC after strong training, home cage, or immediate shock with the same parameters as strong training (5 shocks, 1 mA). Arc expression was significantly greater in the ACC in mice trained with 5 unsignaled foot shocks (5S) compared to home cage controls (HC) and immediate shock controls (IS) (*p* < 0.0001). **D**: Representative images of Arc immunohistochemistry in the ACC. From left to right, a representative image of the 5-shock training, home-cage controls, and immediate shock controls. *p ≤ 0.05; ***p ≤ 0.001; ****p ≤ 0.0001.

**Fig. 2:**
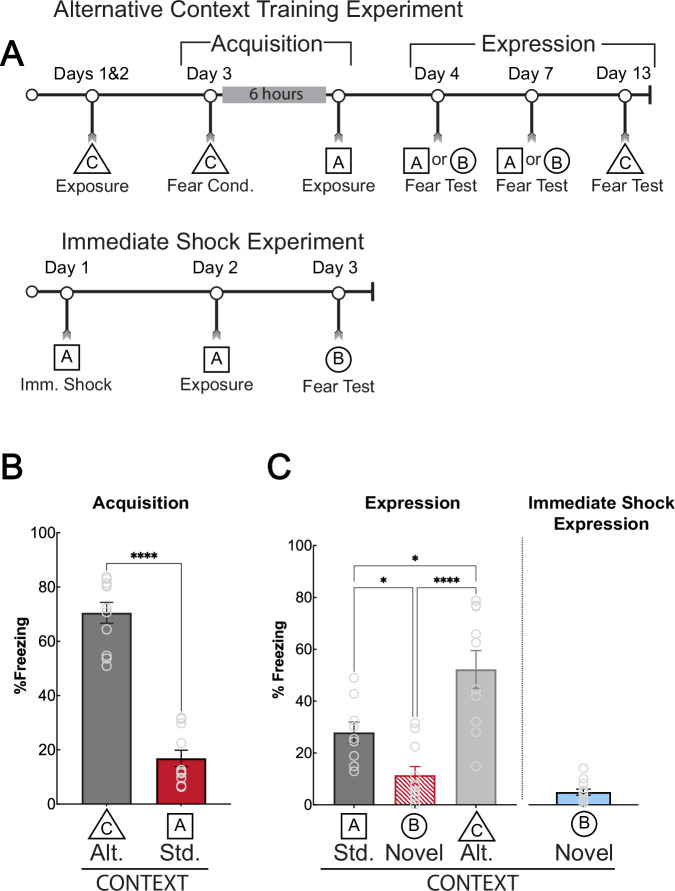
Generalization requires associating a general context representation and threat. **A**: Timeline for the alternative context training procedure and the immediate shock procedure. The boxes below the timeline represent the context in which the mice are placed during each part of the experiment. Mice were fear-conditioned in an alternative training context (triangle, C), then exposed to the standard training context (square, A) six hours later. Mice were then tested for fear in a counterbalanced design in the standard training (square, A) and novel contexts (circle, B). They were then tested in the alternative training context (triangle, C). For the immediate shock procedure, mice were placed in the training context (square, A) and immediately received 5 footshocks and were immediately removed from the context. Twenty-four hours later, mice were exposed to the training context for nine minutes. One day later, mice were tested for fear generalization in a novel context (circle, B). **B**: Acquisition for the alternative context training experiment. Mice display higher freezing in the alt. (alternative, C) training context during training compared to the std (standard, A) training context exposure [t (10) = 10.56, *p* < 0.0001]. **C**: Mice did not generalize fear to the novel context (circle, B) when trained using the alternative training context procedure. Freezing was higher during the test in the standard (std, square, A) training context than in the novel (circle, C) context (*p* = 0.0275), likely due to some transfer of learning during the unpaired context exposure on the acquisition day. There was a significant difference between the standard training context and the alternative training context (*p* = 0.0132), as well as the novel context and the alternative training context (*p* < 0.0001). Freezing in the standard training context did not differ between training and the expression test (*p* = 0.1004). Mice that received immediate shock in the standard training context and tested in a novel context also displayed low freezing, suggesting that generalization is not strictly due to shock exposure without a representation of context. *p ≤ 0.05; ****p ≤ 0.0001.

To investigate the role of the ACC during learning in encoding generalization, we first used Arc expression after different fear conditioning parameters as an indicator of neuroplasticity (Fig. 1A). Arc is a protein involved in learning and memory and is a reliable indication of neuroplasticity [36, 37]. Male and female mice were trained using five un-signaled foot shocks in a contextual fear conditioning procedure, an immediate shock procedure to control for shock experience, or a home cage control procedure. During contextual fear conditioning, mice increased post-shock freezing after successive presentations of the shocks until they reached asymptote after the third shock presentation [F (2.204, 17.63) = 61.68, *p* < 0.0001] (Fig. 1B). Arc staining in the ACC was significantly greater following 5 shock context fear training (*N* = 9) compared to immediate shock (*N* = 4) and home-cage control (*N* = 7) groups [F (2, 17) = 53.62, *p* < 0.0001] (Fig. 1C, D). Next, we trained male and female mice in a distinctly different context to demonstrate that fear generalization depends on associative learning between the shock and the similarities of the contextual elements that are presented during training and testing [38, 39]. Mice (*N* = 11) were trained in the context fear conditioning procedure described above but in an alternative training context (Context C) with different cues (Med Associates 8.25″ W × 7.5″ D × 11″ H) and no illumination. Six hours after training, they were placed into the standard training context (Context A), which served as an ‘unpaired’ context for nine minutes (Fig. 2A). The average freezing was significantly higher in the alternative training context (Context C) compared to the ‘unpaired’ context (Context A), as expected [t (10) = 10.56, *p* < 0.0001] (Fig. 2B). Mice were tested for fear expression in the ‘unpaired’ context (Context A) and a novel context (Context B) in a counterbalanced design. After the final context test, mice were returned to the alternative training context (Context C) six days later (Day 13) to assess freezing to the context in which they were trained (Fig. 2A, C). There was a significant effect of context [F (1.915, 16.28) = 27.70, *p* < 0.0001]; during fear expression tests, mice displayed significantly higher freezing in the alternative training context (Context C) compared to the unpaired (Context A) and novel (Context B) contexts (Tukey’s MC test *p* = 0.0132; *p* < 0.0001). To demonstrate that shock exposure alone does not produce generalization and requires some representation of the training context, we utilized an immediate shock procedure. Mice (*N* = 12) were exposed to the immediate shock procedure as previously described. Twenty-four hours later mice were returned to the training context where they received a 9-min context exposure. The next day they were tested for fear expression to the novel context. All mice showed low freezing to the novel context, indicating no context generalization (Fig. 2C). Overall, these experiments demonstrate that shock alone during the strong training procedure is not sufficient to produce generalization and suggest that associative learning between the shock and the similarities of the contextual elements present during training and testing is necessary for generalized learning.

### NMDAR-dependent mechanisms in the ACC are necessary to encode general information about a specific contextual fear experience to support generalization

**Fig. 3:**
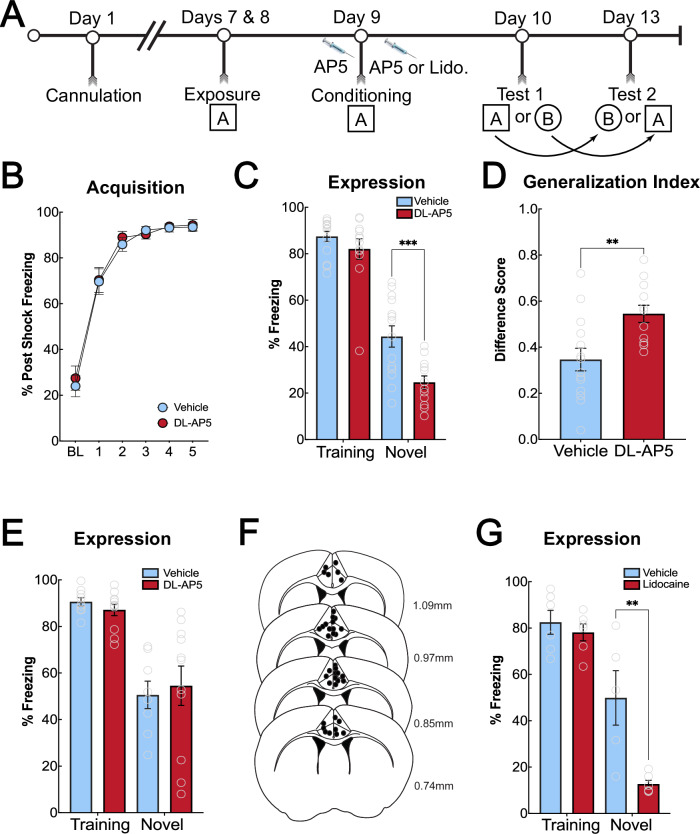
Inactivation of NMDAR in the ACC blocks the acquisition of generalized context fear. **A**: Timeline of behavioral experiments. **B**: Acquisition of context fear conditioning during the experiment in which mice were infused with DL-AP5 before training. There were no differences between DL-AP5 and vehicle-treated mice during acquisition (*p* = 0.7049). **C**: NMDAR blockade in the ACC during learning significantly attenuates contextual fear generalization. Mice receiving DL-AP5 during training displayed significantly less freezing to the novel context than vehicle-treated mice (*p* = 0.0008). Freezing to the training context was not different between the groups (*p* = 0.5155). **D**: Generalization index for pre-training DL-AP5. AP5-treated mice had a significantly greater difference score compared to vehicle-treated mice, indicating a greater difference between their training context freezing compared to their novel context freezing [t (24) = 3.14, *p* = 0.0044]. **E**: Post-training NMDAR blockade in the ACC does not affect context fear generalization or context-specific fear. There were no significant differences in freezing between AP5- and vehicle-treated mice in the novel (*p* = 0.9629) or training context (*p* = 0.9719). **F**: Schematic of cannula placements in the ACC. Placements for pre-training and post-training DL-AP5 experiments are combined into one schematic. **G**: Post-training inactivation of the ACC with lidocaine blocked context fear generalization but had no effect on context-specific fear. Mice infused with lidocaine immediately after training showed a significant reduction in freezing to the novel context compared to vehicle-treated mice (*p* = 0.0025). There were no differences in freezing in the training context (*p* = 0.9531). **p ≤ 0.01; ***p ≤ 0.001.

To further test the idea that associative plasticity in the ACC during fear learning accounts for generalized learning, we utilized the competitive NMDA receptor antagonist, DL-AP5. Male and female mice (*N* = 29) received DL-AP5 or vehicle immediately before undergoing context fear conditioning (Fig. 3A). One mouse was removed as a behavioral outlier, and two mice were removed due to missed targets, leaving *N* = 12 (DL-AP5) and *N* = 14 (Vehicle). We found no significant difference between treatment groups during acquisition [F (1, 25) = 0.1467, *p* = 0.7049] (Fig. 3B). There were no differences in fear acquisition or expression in the training or novel contexts between male and female mice (Figure S1, 3-way ANOVA no main effect of sex, F(1, 22) = 2.385, *p* = 0.14). Therefore, all data was collapsed across sex. During the drug-free fear recall tests, mice that were administered AP5 during training displayed significantly less freezing in the novel context compared to mice administered vehicle control. AP5 had no effect on freezing in the training context (main effect of treatment [F (1, 24) = 9.660, *p* = 0.0048]; main effect of context [F (1, 24) = 243.4, *p* < 0.0001]; significant interaction effect [F (1, 24) = 4.986, *p* = 0.0351]; Sidak’s Novel Context *p* = 0.0008; Sidak’s Training Context *p* = 0.5155) (Fig. 3C). We calculated freezing difference scores between the training and novel contexts using the formula [novel context freezing – training context freezing] / [novel context freezing + training context freezing] as a generalization index. AP5-treated mice had a significantly reduced generalization index (i.e., greater freezing difference between the training and novel context) compared to vehicle-treated mice [t (24) = 3.14, *p* = 0.0044] (Fig. 3D), indicating reduced generalization. Given that NMDA receptors are necessary for long-term potentiation and associative learning [40], these data suggest associative plasticity in the ACC is necessary for mice to encode general contextual elements that promote generalized fear during recall tests in a novel context. Importantly, post-training infusions of AP5 had no effect on freezing in either context (main effect of context only [F (1, 35) = 41.01, *p* < 0.0001; Tukey’s HSD, *p* = 0.96] (*N* = 45; DL-AP5: *N* = 22, Vehicle: *N* = 17, 6 mice removed due to missed targets) (Fig. 3E), consistent with data suggesting that NMDA receptors are only necessary for the induction of LTP and not its maintenance [41, 42]. It is important to point out that NMDAR blockade did not affect learning to the specific training context, suggesting that the role of NMDAR-dependent plasticity in the ACC is to encode general information that supports memory generalization.

### The ACC consolidates general information about a specific contextual fear experience to facilitate memory generalization

Given that NMDAR-dependent plasticity in the ACC is required to acquire a generalized fear memory, we next wanted to determine if the ACC was involved in consolidating newly acquired generalized memories. To accomplish this, we used a post-training manipulation, which is typically thought to influence the consolidation of memory [43–45]. Male mice (*N* = 24; Lidocaine: *N* = 12, Vehicle: *N* = 11, One mouse removed for a missed target) were trained in the context fear task as above and received lidocaine immediately after training to reversibly inactivate the ACC (Fig. 3A). Twenty-four hours after training, mice were tested in the training or novel contexts. Post-training lidocaine infusions in the ACC eliminated generalized fear to the novel context but left specific fear to the training context intact (main effect of context [F (1, 19) = 64.52, *p* < 0.0001], main effect of treatment [F (1, 19) = 11.58, *p* = 0.0030], significant interaction [F (1, 19) = 7.206, *p* = 0.0147], Tukey’s HSD, (*p* = 0.0025) (Fig. 3G). These experiments demonstrate that the ACC is critical for encoding and consolidating generalized context memory associated with elevated threat. The ACC, however, is not necessary to acquire or consolidate specific contextual memory of the training context, at least under the training conditions used here.

### Generalized contextual memory formation requires the ACC, but not the PL

**Fig. 4:**
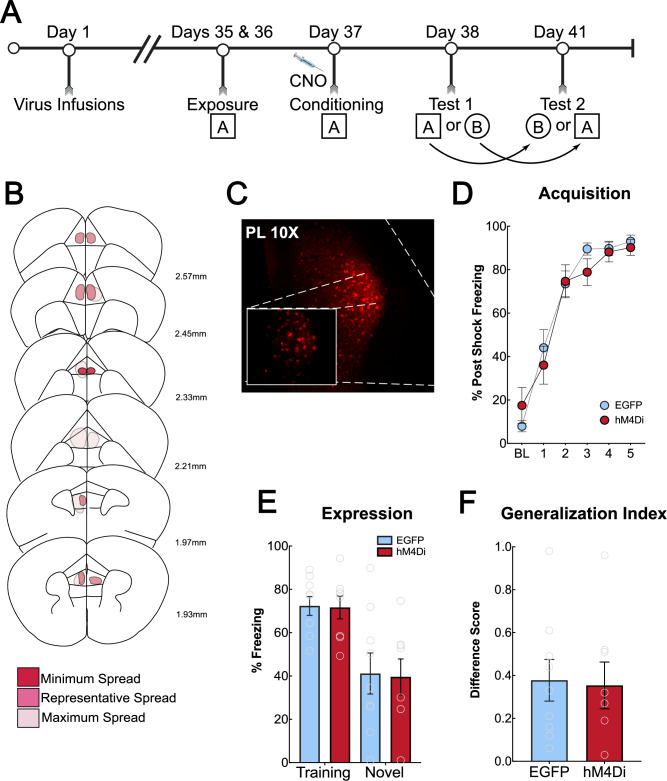
Chemogenetic inactivation of the prelimbic cortex during training does not reduce context fear generalization. **A**: Timeline of behavioral experiment. Mice were infused with an hM4Di-mCherry or EGFP-expressing control AAV and received injections of CNO before context fear conditioning. Mice were tested for fear responses in the training (square, A) and novel (circle, B) contexts in a counterbalanced design. **B**: Schematic representation of hM4Di-mCherry expression in the prelimbic cortex (PL). Dark pink represents minimum virus spread, medium pink represents average, and light pink represents maximum virus spread. **C**: Representative image of PL hM4Di-mCherry expression. **D**: During acquisition, mice expressing hM4Di or EGFP showed no differences in their post-shock freezing (main effect of treatment; *p* = 0.7150). **E**: Mice were tested for fear expression in the training and novel context with 72-h between tests. There were no differences in freezing in the training (*p* = 0.9970) or novel context (*p* = 0.9854) between hM4Di- and EGFP-expressing mice. **F**: Generalization index for PL inactivation. There were no differences in the generalization index between hM4Di-expressing and EGFP -expressing mice (*p* = 0.8709).

We were next interested in understanding if the ACC plays a unique role in regulating generalized contextual memory formation or if this process involves multiple prefrontal cortical regions. We investigated the prelimbic cortex (PL; A32) given its role in fear expression [46, 47] as well as regulating discrimination between aversive and non-aversive cues [10]. Male and female mice (*N* = 19) received bilateral infusions of inhibitory DREADD virus hM4Di or EGFP (*N* = 8 hM4Di, *N* = 9 EGFP; 2 mice were removed due to high baseline freezing prior to shock). Five weeks after surgery, mice underwent contextual fear conditioning using the abovementioned procedure (Fig. 4A). Viral spread analysis and representative images are presented in Fig. 4B, C. Thirty minutes prior to training, all mice received an i.p injection of CNO to inactivate the PL. There was no difference in acquisition between the groups (main effect of shock only [F (2.981, 44.71) = 95.18, *p* < 0.0001], Fig. 4D). Mice received counterbalanced tests in the training and novel contexts. We found a significant main effect of context [F (1, 15) = 23.55, *p* = 0.0002]. However, Sidak’s post-hoc analysis did not reveal a significant difference between hM4Di or EGFP for the training (*p* = 0.9970) or novel (*p* = 0.9854) contexts (Fig. 4E). The generalization index was also not different between the groups [t (15) = 0.1653, *p* = 0.8709] (Fig. 4F). This emphasizes that the ACC is important for regulating generalized contextual memory formation and is not likely controlled by the PL. However, the PL and infralimbic cortex (IL) are implicated in explicit cue generalization [10, 13, 34]. Recruitment of these regions may rely on more explicit cues being present (e.g., tones) compared to more ambiguous and diffuse contextual cues.

### BLA projections to the ACC are necessary for encoding general threat information to promote generalized context fear

**Fig. 5:**
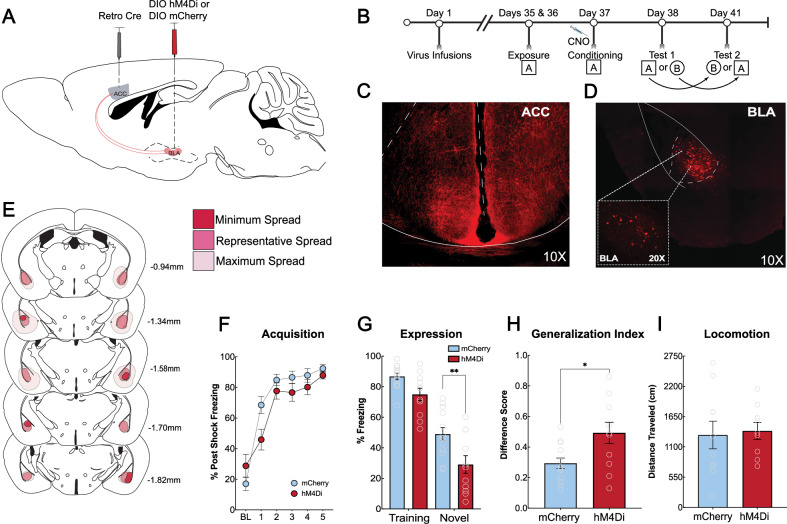
BLA Inputs to the ACC are necessary to encode context fear generalization. **A**: Schematic of viral inactivation strategy. A retrograde AAV expressing cre recombinase was bilaterally injected into the ACC and a cre-dependent AAV expressing hM4Di or mCherry (DIO hM4Di-mCherry or DIO mCherry) was bilaterally injected into the BLA. This enabled specific inactivation of BLA neurons projecting to the ACC. **B**: Timeline of the behavioral experiment. After viral infusions, mice underwent context fear conditioning and were tested for fear responses in the training (square, A) and novel (circle, B) contexts in a counterbalanced design. **C**: Representative image of fiber expression in the ACC. A 10X confocal image was acquired. Image shows hM4Di-mCherry fiber expression in the ACC demonstrating fibers from BLA projection neurons. **D**: Representative image of hM4Di-mCherry expression in the BLA. Images were obtained on a stellaris confocal microscope with a 10x objective (main image). Inset is a 20x image of neurons within the BLA. **E**: Schematic of hM4Di viral spread in the BLA. Dark pink represents minimum virus spread, medium pink represents average, and light pink represents maximum virus spread. **F**: Inactivation of BLA-to-ACC circuit during learning. All mice showed increased post-shock freezing with each shock delivery and there were differences between the groups (main effect of treatment; *p* = 0.1379). **G**: Inactivation of the BLA-to-ACC circuit during context fear learning is necessary for context fear generalization. BLA-to-ACC circuit inactivation eliminated generalization in the novel context, but specific fear to the training context was unaffected (main effect of context [F (1, 22) = 9.014, *p* = 0.0066] Sidak’s post hoc, *p* = 0.0031). **H**: Generalization index for hM4Di-expressing and mCherry-expressing control mice. hM4Di-expressing mice had greater freezing difference scores between the training and novel context compared to mCherry-expressing controls [t (22) = 2.693, *p* = 0.0133]. **I**: Inactivation of BLA-to-ACC circuit did not alter locomotion in the open field. Mice received i.p. injections of CNO (5 mg/kg) 30 min prior to being placed in the open field. We saw no significant difference in distance traveled between hm4Di- and mCherry-expressing mice [t (17) = 0.2473, *p* = 0.8076]. *p ≤ 0.05; **p ≤ 0.01.

We next investigated circuit mechanisms underlying how the ACC encodes generalized fear memories. For the ACC to encode and consolidate generalized memories, it would likely need to receive threat information from the amygdala. While additional regions could provide contextual or nociceptive information such as the hippocampus and thalamus, we first chose to investigate the inputs from the basolateral amygdala (BLA), given its critical role in fear acquisition, consolidation, and expression [48–53]. We utilized an intersectional chemogenetic approach bilaterally injecting a retrograde cre-expressing virus in the ACC (pAAV-Ef1a -cre (AAVrg)) and a cre-dependent virus encoding hM4Di or mCherry bilaterally in the BLA (pAAV-hSyn-DIO-hM4D(Gi)-mCherry (AAV8) or pAAV-hSyn-DIO-mCherry (AAV8)) (Total *N* = 36; *N* = 11 hM4Di, *N* = 13 mCherry, 3 mice removed for unilateral hits, 9 removed for misses) (Fig. 5A). Five weeks after surgery, mice underwent contextual fear conditioning. Thirty minutes prior to training, all mice received an i.p injection of CNO (Fig. 5B). Images of viral expression BLA and ACC, as well as spread analysis are represented in Fig. 5C, D, E. Male and female mice injected with the hM4Di- or mCherry-expressing virus acquired fear similarly (Fig. 5F). Mice were tested in the training or novel context in a counterbalanced design with 72 h in between tests. BLA-to-ACC circuit inactivation eliminated generalization in a novel context, but specific fear to the training context was unaffected (main effect of treatment [F (1, 22) = 9.014, *p* = 0.0066]).We found no differences between males and females; therefore, the data were collapsed and analyzed together (3-way ANOVA; main effect of context [F(1, 20) = 249.4, *p* < 0.0001]; main effect of virus [F(1, 20) = 7.535, *p* = 0.0125], no main effect of sex [F(1, 20) = 3.472, *p* = 0.0772], Figure S2). Post-hoc analysis revealed a significant difference between hM4Di and mCherry groups for freezing in the novel context (*p* = 0.0031) (Fig. 5G). hM4Di-expressing mice also had a significantly reduced generalization index (i.e., higher freezing difference score compared to mCherry controls) [t (22) = 2.693, *p* = 0.0133] (Fig. 5H), suggesting reduced generalization. To verify that inactivation of the BLA-to-ACC circuit did not alter locomotor behavior, we assessed the inactivation of this circuit on locomotion using an open field. Mice received i.p. injections of CNO 30 min prior to being placed in the open field. There was no significant difference in distance traveled between mCherry and hM4Di expressing mice [t (17) = 0.2473, *p* = 0.8076] (Fig. 5I). We also saw no differences for time spent in the center [t (17) = 0.8396, *p* = 0.4128], indicating a lack of effect on anxiety-like behavior. These results indicate that the BLA’s inputs to the ACC during learning, are critical in providing threat information that is utilized by the ACC to encode and consolidate generalized contextual fear memories under increased levels of threat.

### The strength of threat information from the BLA-to-ACC pathway determines the encoding of context fear generalization

**Fig. 6:**
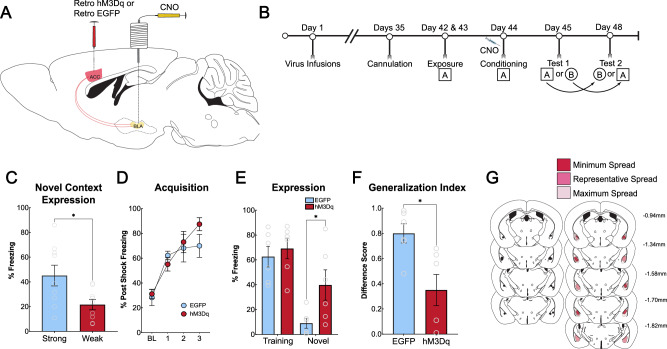
BLA inputs to the ACC drive context fear generalization under training conditions that normally do not support generalization. **A**: Schematic of circuit activation strategy. A retrograde AAV expressing hM3Dq-mCherry or EGFP was bilaterally injected into the ACC. Five weeks later, mice were bilaterally cannulated over the BLA to enable local infusions of CNO in the BLA. One week after cannulations, mice underwent fear conditioning. Infusions of CNO via guide cannula enabled direct activation of BLA neurons that project to the ACC. **B**: Timeline of behavioral experiments. Mice received a bilateral infusion of CNO into the BLA immediately before undergoing context fear training. They were then tested in the training and novel context in a counterbalanced design. **C**: Mice were trained with either a three-shock, 0.6 mA protocol (weak training) or a 5 shock, 1.0 mA (strong training). There was a significant difference in freezing to the novel context (*p* = 0.0233). Mice trained with weak fear conditioning displayed less freezing to the novel context than mice with strong training. Mice that underwent the strong training procedure displayed the expected generalization to the novel context. **D**: When the BLA-ACC circuit was activated using chemogenetics during training, both groups of mice acquired context fear as expected, with no differences between treatment groups (main effect of shock [F (2.289, 22.89) = 18.00, *p* < 0.0001]). **E**: During a fear expression test, hM3Dq-expressing mice froze significantly more in the novel context compared to EGFP-expressing mice (main effect of context [F (1, 10) = 43.02, *p* < 0.0001], Sidak’s post-hoc (*p* = 0.0410)). **F**: The generalization index indicated a greater difference in freezing between the training context and novel context in EGFP-expressing mice compared to hM3Dq-expressing mice [t (10) = 3.089, *p* = 0.0115]. **G**: Schematic of cannula placements in the BLA and hM3Dq-mCherry in the BLA. *p ≤ 0.05.

We next wanted to test if threat information carried by BLA projections to the ACC could drive encoding of generalization under weak training conditions. Given that we did not identify sex differences in the above-mentioned studies, we utilized male mice in this experiment. First, we ran naïve mice through a weak training protocol consisting of 3 un-signaled foot shocks delivered at 0.6 mA (*N* = 10, 1 mouse removed as a behavioral outlier leaving *N* = 9), or the strong training protocol (*N* = 9) used earlier. Twenty-four hours following training, mice were tested in a novel context to assess context generalization. Mice that received strong training (5 shocks, 1.0 mA) froze significantly higher compared to mice exposed to the weak training protocol (3 shocks, 0.6 mA) [t (16) = 2.508, *p* = 0.0233] (Fig. 6C), indicating that mice trained using the strong training protocol generalized fear to the novel context, whereas the mice trained using the weak protocol did not. Therefore, the weak training parameters were used to test the sufficiency of the BLA’s inputs to the ACC during context fear learning. Mice (*N* = 19) were injected with a retrograde virus in the ACC that expressed the chemogenetic excitatory receptor, hM3Dq, or a control virus encoding EGFP (pAAV-hSyn-hM3Dq-mCherry (AAVrg); pAAV-CaMKIIa-EGFP (AAVrg)) (*N* = 6 hM3Dq, *N* = 6 EGFP, 3 mice removed for misses, 1 for unilateral virus expression, 2 removed for lack of infusion 1 removed for lack of shocks during training) (Fig. 6A). Five weeks later, mice were bilaterally cannulated over the BLA to enable infusions of CNO to locally activate ACC-projecting BLA neurons. Following an additional week of recovery, mice were run through context fear conditioning utilizing the weak training protocol. Five minutes prior to training, all mice received infusions of 0.2 μL/hemisphere of CNO into the BLA [5] (Fig. 6B). There were no differences in fear acquisition between the treatment groups (main effect of shock [F (2.289, 22.89) = 18.00, *p* < 0.0001]) (Fig. 6D). Twenty-four and seventy-two hours after training, mice were tested in the training and novel contexts in a counterbalanced design. Mice expressing hM3Dq froze significantly more in the novel context compared to those expressing EGFP [Sidak’s post-hoc (*p* = 0.0410)] (Fig. 6E). The generalization index also showed the same effect with hM3Dq-expressing mice displaying more generalization compared to EGFP-expressing mice [t (10) = 3.089, *p* = 0.0115] (Fig. 6F). Viral spread analysis is represented in Fig. 6G. Collectively, these results suggest that inputs from the BLA to the ACC convey threat information, and activation of this input is critical for mice to encode general information regarding a threatening experience. This pathway is likely part of a larger circuit that is recruited to enable animals to generalize responses to novel environments, which in turn allows animals exposed to highly threatening experiences to quickly assess similar situations for the likelihood of threat and respond with appropriate defensive behavior.

## Discussion

In rodent fear literature, the ACC has been best characterized for its role in remote contextual fear [17–20, 54, 55]. However, we and others have consistently shown a role for the ACC in regulating generalized fear expression both at remote and recent testing periods after conditioning [5, 15–17]. Clinical investigations have found a role for both the ACC and the BLA in anxiety and stress-related disorders [56–59]. The ACC within these studies is analogous to the human midcingulate cortex, often referred to as just anterior cingulate cortex within clinical studies [60]. Additionally, hyperfunction of the ACC and BLA has been shown to be a predisposition for developing PTSD [59], and overgeneralization of fear is a core symptom of this disorder [61–63]. Our findings show that the ACC is recruited during training, where Arc activity was highest compared to our control groups (Fig. 1C). Next, we identified that NMDAR-dependent plasticity is necessary, which we hypothesize is evoked by inputs from the BLA, to allow for generalized fear expression 24 h after fear learning. Combined with the post-training inactivation results, our data suggests that the ACC consolidates a general representation of the training experience to guide fear in novel environments (Fig. 3). Finally, we found that BLA-to-ACC inputs are both necessary and sufficient for encoding generalized representations (Figs. 5 and 6).

To decipher the role of the ACC in processing generalized information, we first investigated its activity following strong training, which produces generalized fear responses [5]. The ACC has been implicated in processing pain information [64–68], therefore we utilized an immediate shock procedure, using the same shock parameters as our strong training protocol [28, 29], to demonstrate that shock alone is not sufficient to drive ACC activity and fear generalization. When we measured ACC Arc protein expression, we saw that the immediate shock group had significantly fewer Arc-expressing cells compared to the strong training group (Fig. 1C), however, mice that underwent immediate shock showed significantly more Arc expression than home cage controls in the ACC as would be expected because of the ACC’s role in pain processing. Mice exposed to immediate shock did not generalize fear to the novel context, emphasizing that strong shock exposure alone does not produce generalization. Using an unpaired training procedure in which mice received shocks and context exposure separately, we further showed that nociceptive pain processing alone is not sufficient to drive context generalization (Fig. 2C).

Next, we found that inactivation of NMDA receptors in the ACC prior to learning significantly reduced fear in the novel context but left fear in the training context intact (Fig. 3C). Post-training infusions of AP5 had no effect on generalized fear (Fig. 3E). Long-term potentiation is thought to be the basis of consolidation of memories [40]. NMDA receptors are generally necessary for the induction of plasticity but not for its maintenance across time [41, 42]. These data suggest that the mechanisms within the ACC that drive generalized context fear are mediated through NMDAR-dependent synaptic plasticity. Furthermore, immediate post-training inactivation of the ACC significantly attenuated context fear generalization, suggesting that cellular activity within the ACC following learning is critical for generalization (Fig. 3G). We next verified that our results are restricted to the ACC, and not the PL, given its role in fear expression [46, 47] as well as discriminating between aversive and non-aversive cues [10]. Inactivation of the PL prior to strong training did not affect the acquisition (Fig. 4D) or expression of fear in the training or novel context (Fig. 4E). These data replicate previous findings that show pre-training inactivation of the PL does not alter context fear expression [46]. This previous report did, however, see reduced freezing during training after PL inactivation with TTX, which we did not observe here using DREADD inactivation and a strong training procedure. The current data, as well as previous reports support the idea that the ACC is recruited to encode and consolidate generalized fear memories, which are not reliant on the PL.

Whereas we consistently observe a selective role for the ACC in regulating generalized context fear, several previous studies have demonstrated that the ACC is important for consolidating or expressing fear in the training context. It is difficult to reconcile the differences among these studies and why some have found a selective role for the ACC in generalized context memory [5, 15, 17], and others have found it to regulate consolidation and expression of specific context memory [18, 19, 69]. The discrepancies among these studies could be related to the strength and type of training used in each study.

The BLA is known for its role in both the acquisition and expression of fear [48–53, 70], making it a critical node for both receiving and sending information about a threatening experience. The BLA and ACC share reciprocal connections [23, 71], and we have previously identified that ACC efferent projections to the BLA regulate generalized context fear expression [5]. Here, we investigated the role of the ACC itself and ascending BLA projections to the ACC during context fear learning. Before DREADD experiments, we trained surgical naive mice using contextual fear conditioning and administered CNO before learning (*N* = 11 CNO, *N* = 11 vehicle). We found that CNO alone, without DREADD virus expression, did not affect fear learning (Figure S3B) or fear recall in both the training and novel context and did not affect locomotion (Figure S3C). When we inactivated the BLA-ACC circuit, we found a significant reduction of freezing in the novel context but not the training context (Fig. 5G), suggesting that BLA-to-ACC projections are critical in promoting the encoding of a generalized fear memory. In contrast, activating BLA inputs to the ACC induced generalization to the novel context (Fig. 6E). Taken together these data indicate that the BLA-to-ACC circuit is both necessary and sufficient for encoding context fear generalization. The amygdala is thought to assign positive and negative valence [72, 73] to events that evoke strong emotional responses, such as fear [74, 75]. In contrast, the ACC is implicated in regulating action selection [76–78] and decision-making [79]. Therefore, inputs from the BLA to the ACC likely convey threat information associated with aversive stimuli that engage the ACC to initiate adaptive behavioral strategies in uncertain situations, such as placement into a novel context.

Our procedure utilized two days of pre-exposure, which allows the animals to form a robust hippocampal representation of the training context [29, 80]. Therefore, the generalization we observe in the novel context is unlikely to be the result of a poor hippocampal representation of the training context, as would be expected if the mice were not pre-exposed for several minutes [81]. The cortex processes contextual information [82–85], and in the absence of a precise hippocampal memory, it has been suggested that an impoverished representation is formed [22]. Therefore, the ACC could be a region where an impoverished context representation can be encoded after high threat levels that would facilitate generalized responding across novel but similar contexts. In this case, because mice in these experiments have an intact hippocampus, this would suggest that both a contextually precise memory dependent on the hippocampus and an impoverished or generalized memory dependent on the ACC exist simultaneously. The determination of which memory is expressed could rely on the test parameters.

BLA-to-ACC inputs have been shown to become active following pain, as well as being linked to pain-induced depressive-like behaviors [86]. Therefore, plasticity in the ACC may be partially evoked by pain during context fear learning. However, the Arc expression suggests that the ACC is more active following context fear conditioning than immediate shock, which represents the activity of the shock alone without an association (Fig. 1). In addition, with weak conditioning, mice fail to generalize fear the following day (Fig. 6). However, in the presence of strong training, BLA inputs to the ACC drive threat-related plasticity. It is important to note that thalamic subregions send projections to the ACC [23], and it has recently been identified that medial dorsal thalamus-to-ACC projections evoke pain-related avoidance [87] as well as alterations in synaptic plasticity on anterior thalamic projections to the ACC following neuropathic pain [88]. Therefore, the thalamus could mediate pain-induced plasticity, which is strengthened by recruiting the BLA inputs to the ACC to allow NMDAR-dependent encoding.

In sum, our findings suggest that the ACC may be an integration site for encoding threat information from the amygdala to facilitate generalized behavioral responses. Given that our tests involve shifts in context, we would expect a role for the ventral hippocampus in this process [5, 12, 15]. However, an interaction between the ventral hippocampus and ACC has not yet been demonstrated in intensity-dependent generalization. The ACC, BLA, and vHPC likely act as a coordinated circuit to contribute to learning and generalizing within the ACC, enabling animals to predict threatening environments and improve survival. Our findings did not reveal sex divergent responses, suggesting that the mechanisms for encoding threat-induced fear generalization are conserved across sexes using these training parameters [89]. Overall, the ACC might integrate information received from several areas to promote flexible behavioral responses following learning. These responses, depending on the context, could be a product of schema or categorical learning, both of which are known to involve the cortex [90–92] which enable animals to respond adaptively to changing environments after highly salient events. Understanding the circuitry and the role of the ACC in promoting this behavioral adaptation creates potential avenues for developing therapeutics for individuals who fail to flexibly respond to their changing environments, and thus has therapeutic potential for anxiety and/or stress-related disorders [93].

## Supplementary information

Suplemental Material File

## Data Availability

The datasets generated and analyzed for the current study are available from the corresponding author on reasonable request.
