# Supplementary material for: An amygdala-cortical circuit for encoding generalized fear memories
Source: Mol Psychiatry. 2025 Aug 12;31(1):430–43. doi: 10.1038/s41380-025-03140-8 (PMC12700787; doi:10.1038/s41380-025-03140-8)
Supplement: Supplementary file 1 — Suplemental Material File [file 41380_2025_3140_MOESM1_ESM.pdf]

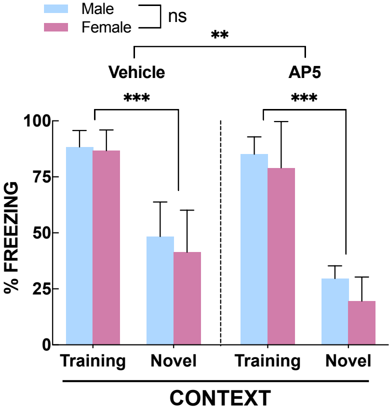

Supplemental Figure 1: No Sex Differences in NMDAR Inactivation on Fear Generalization. Fear expression test in male and female mice. A 3-way ANOVA (Context x Treatment x Sex) indicated there was a main effect of context ($F(1, 22) = 224.4$; $P<0.0001$), a main effect of treatment (AP5 v. vehicle) ($F(1, 22) = 10.26$; $P=0.0041$) and a context x treatment interaction ($F(1, 22) = 4.878$; $P=0.0379$). There was no main effect of sex ($F(1, 22) = 2.385$; $P=0.1368$) and no additional significant interactions.

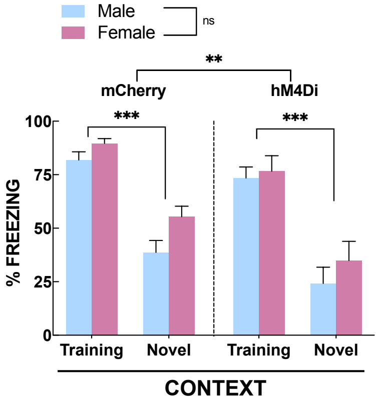

Supplemental Figure 2: No Sex Differences in BLA-ACC Inactivation on Fear Generalization. Fear expression test in male and female mice. A 3-way ANOVA (Context x Treatment x Sex) indicated there was a main effect of context [$F(1, 20) = 249.4$, $p < 0.0001$]; a main effect of virus [$F(1, 20) = 7.535$, $p = 0.0125$], but no main effect of sex [$F(1, 20) = 3.472$, $p = 0.0772$].

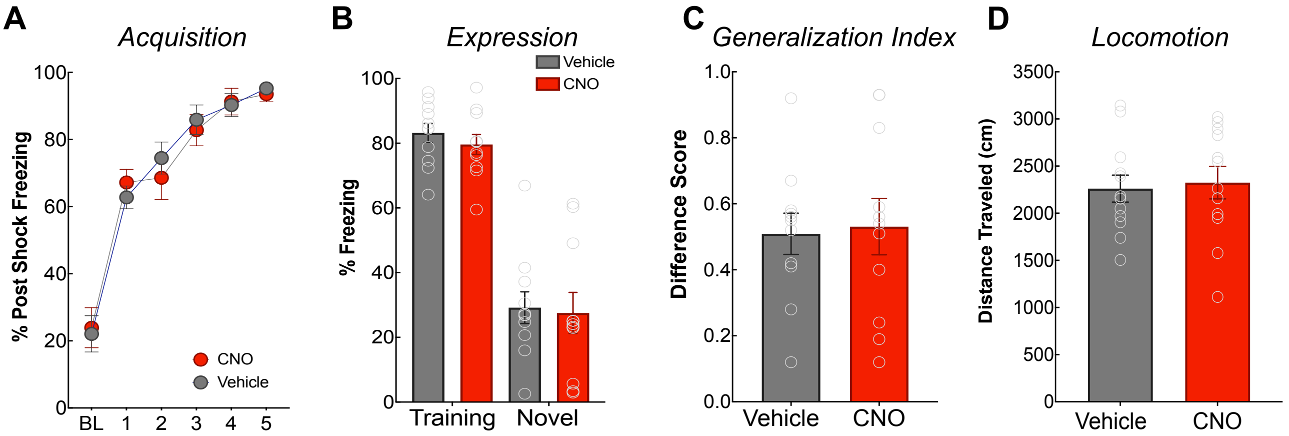

Supplemental Figure 3: CNO itself has no effect on fear learning, recall, fear generalization or locomotion. Mice were injected i.p. with CNO (5 mg/kg) or vehicle (0.9% sterile saline) 30 minutes before fear conditioning. Mice were fear conditioned with 5 unsignaled footshocks (1 mA) idenditcal to the procedure used for experiments described in the manuscript. **A)** There was no difference between CNO- and vehicle-treated mice in fear acquisition. **B)** Fear expression test in the training context or the novel context. There was no difference in freezing in the training ($p = 0.9598$) or novel ($p = 0.8232$) context between CNO- and vehicle-treated mice. **C)** The generalization index was not different between CNO- and vehicle-treated mice [$t\ (20) = 0.2064$, $p = 0.8386$]. D) Mice were injected with CNO i.p. 30 minutes before being placed in an open field and their locomotion was recorded for 10 mintes. There was no difference between CNO- and vehicle-treated mice in distance traveled [$t\ (22) = 0.2843$, $p = 0.7789$].

Analysis	Experiment	Comparison	F/t Statistic	p Value	DF	$\eta p2$	Effect Size	Power	Post-Hoc Test	Figure #
One-Way RM ANOVA	Arc in the ACC	Acquisition	61.68	<0.0001	5	0.86	2.78	1	Tukey's	1B
		Arc Expression	53.62	<0.0001	2	0.86	2.51	1	Tukey's	1C
Paired t-Test	Unpaired Context Fear	Acquisition	10.56	<0.0001	10	–	3.18	1	–	2B
One-Way RM ANOVA		Expression Test	27.7	<0.0001	2	0.41	0.84	1	Tukey's	2C
Two-Way RM ANOVA	Pre-Training DL-AP5	Interaction	0.2	0.9619	5	0.01	0.09	0.19	Sidak's	3B
		Shocks	152.4	<0.0001	5	0.86	2.47	1	Sidak's	
		Treatment	0.1467	0.7049	1	0.004	0.06	0.07	Sidak's	
		Interaction	4.986	0.0351	1	0.17	0.46	0.99	Sidak's	3C
		Context	243.4	<0.0001	1	0.91	3.18	1	Sidak's	
		Treatment	9.66	0.0048	1	0.39	0.8	0.99	Sidak's	
Unpaired t-Test		Generalization Ratio	3.14	0.0044	24	–	1.25	0.86	–	3D
Two-Way ANOVA	Post-Training DL-AP5	Interaction	0.4203	0.521	1	0.01	0.11	0.1	Tukey's	3E
		Context	41.01	<0.0001	1	0.54	1.08	1	Tukey's	
		Treatment	0.002	0.9663	1	0.00005	0.007	0.05	Tukey's	
Two-Way ANOVA	Post-Training Lidocaine	Interaction	7.206	0.0147	1	0.27	0.62	0.8	Tukey's	3G
		Context	64.52	<0.0001	1	0.77	1.84	1	Tukey's	
		Treatment	11.58	0.003	1	0.38	0.78	0.94	Tukey's	
Two-Way RM ANOVA	Prelimbic Cortex Inactivation	Interaction	1.193	0.321	5	0.07	0.28	0.87	Tukey's	4D
		Shocks	95.18	<0.0001	5	0.86	2.52	1	Tukey's	
		Virus Expression	0.1385	0.715	1	0.008	0.09	0.07	Tukey's	
		Interaction	0.004	0.9484	1	0.0003	0.02	0.05	Sidak's	4E
		Context	23.55	0.0002	1	0.61	1.25	1	Sidak's	
		Virus Expression	0.02084	0.8871	1	0.002	0.04	0.05	Sidak's	
Unpaired t-Test		Generalization Ratio	0.1653	0.8709	15	–	0.08	0.05	–	4F

Supplementary Table 1: Statistical Summary for Figures 1-4.

Analysis	Experiment	Comparison	F/t Statistic	p Value	DF	$\eta p2$	Effect Size	Power	Post-Hoc Test	Figure #
Two-Way RM ANOVA	BLa-to-ACC Inactivation	Interation	3.431	0.0064	5	0.13	0.39	1	Sidak's	5F
		Shocks	73.06	<0.0001	5	0.77	1.82	1	Sidak's	
		Virus Expression	2.371	0.1379	1	0.06	0.26	0.36	Sidak's	
		Interation	2.374	0.1377	1	0.1	0.33	0.87	Sidak's	5G
		Context	247.9	<0.0001	1	0.92	3.36	1	Sidak's	
		Virus Expression	9.014	0.0066	1	0.62	1.27	1	Sidak's	
Unpaired t-Test		Generalization Ratio	2.693	0.0133	22	–	1.08	0.71	–	5H
Unpaired t-Test		Distance Traveled	0.2473	0.8076	17	–	0.12	0.06	–	5I
Unpaired t-Test	BLa-to-ACC Activation	Protocol	2.508	0.0233	16	–	1.18	0.65	–	6C
Two-Way RM ANOVA		Interation	1.004	0.4047	3	0.09	0.32	0.68	Tukey's	6D
		Shocks	18	<0.0001	3	0.64	1.34	1	Tukey's	
		Virus Expression	0.8046	0.3908	1	0.03	0.16	0.1	Tukey's	
		Interation	3.699	0.0833	1	0.27	0.61	0.8	Sidak's	6E
		Context	43.02	<0.0001	1	0.81	2.07	1	Sidak's	
		Virus Expression	3.148	0.1064	1	0.47	0.93	0.96	Sidak's	
Unpaired t-Test		Generalization Ratio	3.089	0.0115	10	–	1.78	0.79	–	6F

Supplementary Table 2: Statistical Summary for Figures 5 & 6
